# Kombuchas from Green and Black Tea Modulate the Gut Microbiota and Improve the Intestinal Health of Wistar Rats Fed a High-Fat High-Fructose Diet

**DOI:** 10.3390/nu14245234

**Published:** 2022-12-08

**Authors:** Mirian Aparecida de Campos Costa, Luiza de Paula Dias Moreira, Vinícius da Silva Duarte, Rodrigo Rezende Cardoso, Vinícius Parzanini Brilhante de São José, Bárbara Pereira da Silva, Mariana Grancieri, Viviana Corich, Alessio Giacomini, Josefina Bressan, Hércia Stampini Duarte Martino, Frederico Augusto Ribeiro de Barros

**Affiliations:** 1 Department of Food Technology, Universidade Federal de Viçosa, Avenida Peter Henry Rolfs, s/n, Viçosa 36570-900, MG, Brazil; 2 Department of Agronomy, Food Natural Resources, Animals, and Environment (DAFNAE), Università degli Studi di Padova, Via dell’Università 16, 35020 Legnaro, PD, Italy; 3 Faculty of Chemistry, Biotechnology, and Food Science, The Norwegian University of Life Sciences, P.O. Box 5003, 1432 Ås, Norway; 4 Department of Nutrition and Health, Universidade Federal de Viçosa, Avenida Peter Henry Rolfs, s/n, Viçosa 36570-000, MG, Brazil

**Keywords:** experimental study, gut microbiome, intestinal permeability, obesity, polyphenols, probiotic, short-chain fatty acids

## Abstract

The Western diet can negatively affect the gut microbiota and is associated with metabolic disorders. Kombucha, a tea fermented by a symbiotic culture of bacteria and yeast (SCOBY), is known for its bioactive properties and has become popular in the last years. In this study, we evaluated the effects of regular kombucha consumption on the gut microbiota and on outcomes related to the intestinal health of Wistar rats fed a high-fat high-fructose diet. After eight weeks receiving a standard diet (AIN-93M) (n = 10) or a high-fat and high-fructose diet (HFHF) (n = 30) to induce metabolic disorders, the animals were subdivided into four groups: AIN-93M (n = 10); HFHF (n = 10); GTK (HFHF + green tea kombucha (n = 10); and BTK (HFHF + black tea kombucha; n = 10) for 10 weeks. Although body composition did not differ among the groups, the HFHF diet was associated with metabolic alterations, and stimulated the growth of gram-negative bacteria such as *Proteobacteria* and *Bacteroides*. Kombucha ingestion could somewhat modulate the gut microbiota, attenuating the effects of a Western diet by increasing propionate production and favoring the growth of beneficial bacteria, such as *Adlercreutzia* in the GTK group. Our results suggest that regular kombucha consumption may be beneficial to intestinal health, which can be mostly attributed to its high content and diversity of phenolic compounds.

## 1. Introduction

The Western diet style, despite not having a specific definition, is an unhealthy diet generally composed of a high amount of saturated fat and fructose [1], which is involved in metabolic disorders such as obesity and metabolic syndrome [2,3], as well as in alterations in the gut microbiota profile [4]. Although the mechanisms by which dietary fat can modulate the gut microbiota are not completely understood yet, it is known that the small amount of this nutrient that is not absorbed in the small intestine can be fermented by the gut microbiota. Free fatty acids (FFA) resulting from the lipid metabolism can be utilized as substrates by the microorganisms present in the gut microbiota, influencing its composition [5] by increasing the *Bacteroidetes*:*Firmicutes* ratio [6] and the proportion of *Proteobacteria*, which are a major source of lipopolysaccharides [7]. Similarly, a high-fructose diet has also been linked with alterations in the gut microbiota. Studies have suggested that it can affect the morphology and function of the intestine by altering the structure of the tight junction proteins, leading to increased intestinal permeability and inflammation [8,9].

Kombucha, a fermented beverage usually produced from green or black tea, has been highlighted as a promising alternative to minimize the impact of the Western diet or even for those who wish for a healthier lifestyle [10]. Kombucha presents in its composition several microorganisms as a result of the fermentation process performed by microorganisms known as SCOBY (symbiotic culture of bacteria and yeast) [11,12]. These microorganisms include lactic and acetic bacteria, particularly from the genera *Acetobacter* and *Gluconobacter*, and yeasts [12,13,14,15].

Beyond the microorganisms, kombucha also presents in its composition organic acids such as acetic, gluconic, and glucuronic; vitamins C and B complex; minerals; and amino acids [10,11,16]. However, it seems that the main benefits associated with kombucha intake are due to the presence of bioactive compounds. Previous studies conducted by our research group have shown that kombuchas from green and black tea present a high antioxidant capacity due to a high amount and diversity of phenolic compounds. Of the 127 phenolic compounds that we have identified, 103 were reported for the first time in the literature [17]. Nonetheless, its nutritional composition is influenced by many factors, including the tea type and quality, the amount of substrate, and the time and temperature used in the fermentation process [17,18,19]. SCOBYs also present differences in their composition and can influence the microorganism profile [20,21].

Regardless of the differences obtained in the manufacturing process, kombucha intake has been associated with health benefits through the modulation of the gut microbiota in mice. Black tea kombucha was associated with a decreased abundance of *Allobaculum*, *Turicibacter*, and *Clostridium* genera and an increase in *Mucispirillum*, a genus positively correlated to circulating leptin, which is a hormone involved in the regulation of appetite and food intake [22]. In another study, green tea kombucha supplementation was associated with an increase in alpha-diversity as well as favored the growth of bacteria involved in butyrate production [23]. Additionally, a recent systematic review has pointed out that kombucha consumption was able to reduce intestinal dysbiosis in vivo, being suggested as a potential alternative for the control and treatment of obesity and its associated comorbidities [24].

Although there is evidence that kombucha intake can bring benefits to health, there is still no consensus in the literature, especially when associated with the Western diet. Even though many commercial kombuchas have an appeal as a probiotic product, there is no evidence to support it so far. Thus, we aimed to investigate the effects of regular kombucha consumption on the gut microbiota and on outcomes related to the intestinal health of Wistar rats fed a high-fat high-fructose diet. Based on the chemical and microbiological composition of the beverages, as well as on previous studies [22,23], we hypothesized that green and black tea kombuchas would be able to modulate the gut microbiota and improve the intestinal health of those animals.

## 2. Material and Methods

### 2.1. Kombuchas Preparation

Kombuchas from green and black tea were prepared as previously described [17,25]. In summary, green tea (Lung Ching) and black tea (Darjeeling Gielle FTGFOP1) were obtained in a certified store (Tea Shop^®^) located in Belo Horizonte, Minas Gerais, Brazil.

Both beverages were prepared using 12 g of tea leaves and 50 g of sugar per liter of mineral water. Green tea infusion was performed at 75 °C for 3 min and the black tea at 95 °C for 5 min, according to the manufacturer. The beverages were cooled in an ice bath and when they reached room temperature, they were added to a SCOBY (3% *w*/*v*) (Enziquímica^®^, Gravataí, Brazil). A previously prepared kombucha (10% *v*/*v*) was also added to the beverages to decrease the pH and inhibit the proliferation of pathogenic microorganisms [11].

Fermentation occurred for ten days at 25 °C and then the SCOBY was removed and the kombuchas were filtered (Whatman #1 qualitative filter paper). The beverages were stored at 4°C for up to two weeks before being offered to the animals.

### 2.2. Animal Study

#### 2.2.1. Study Design

Forty Wistar rats (*Rattus norvegicus*) aged between 45 and 50 days old were obtained at the Central Animal Facility from the Center of Biological Sciences and Health at Universidade Federal de Vicosa, Brazil. The animals were allocated to individual stainless-steel cages and kept in a light-dark cycle (12 h/12 h) at room temperature at 22 ± 2 °C.

**Figure 1:**
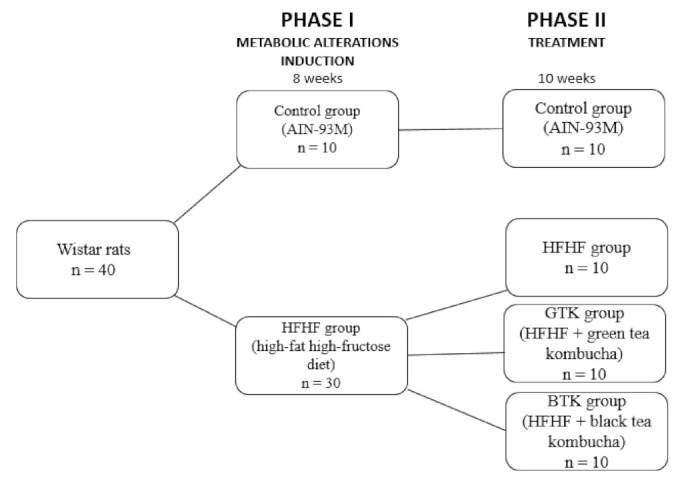
Experimental study design.

The experiment was divided into two phases. Phase I lasted eight weeks and the animals were separated into two groups: group 1 (n = 10); received a standard control diet (AIN-93M) [26] and group 2 (n = 30); received a high-fat and high-fructose (HFHF) diet to induce metabolic alterations [27]. In phase II, which lasted ten weeks, group 1 continued receiving a standard diet while the HFHF group was subdivided into three other groups: HFHF group (positive control) (n = 10); green tea kombucha (GTK group), which received HFHF diet + green tea kombucha diluted in water (30% *v*/*v*) (n = 10); and black tea kombucha (BTK group), which received HFHF diet + black tea kombucha diluted in water (30% *v*/*v*) (n = 10) (Figure 1). Both diets and water were consumed *ad libitum* during the whole experimental period.

All procedures were performed following the ethical principles for animal use in experimental studies. The study protocol was approved by the Ethics Committee on Animal Use (CEUA—Universidade Federal de Vicosa, Protocol 06/2019; date of approval: 28 May 2019).

#### 2.2.2. Kombuchas Characterization and Dosage

This study is a follow-up to our previous work and details about the experimental diet and the physical-chemical analyses of the kombuchas used in this experiment have been extensively described [17,25]. Briefly, sugars (sucrose, glucose, and fructose), organic acids (acetic, glucuronic, and lactic), and ethanol were identified and quantified by high- performance liquid chromatography (HPLC) (Shimadzu, model LC-10A VP) coupled to a refractive index detector (RID 6A). The total acidity was determined according to the methodology proposed by IAL (2008) using phenolphthalein as an indicator and the results expressed as % acetic acid (*w*/*v*) [28]. The pH was measured by a calibrated pH meter (Kasvi, K-39,1014B, China). The concentrations of theaflavins and thearubigins were determined by a spectrophotometer according to the methodology proposed by Jayabalan et al. (2007), and the results were expressed as % (*w*/*v*) [29]. Regarding the microbiological characterization, serial kombucha samples were used to determine acetic bacteria, lactic bacteria, and yeasts using plates with GYC agar and ethanol, MRS, and PDA agar, respectively. The results were expressed as CFU/mL.

Kombucha dosage was determined according to preliminary tests that indicated the total phenolic content of beverages. Calculations were performed based on a previous study that recommends a daily total phenolic intake of 17 mg/kg/body weight [30].

#### 2.2.3. Euthanasia and Samples Collection

As previously described [25], at the end of the experimental period, the animals were anesthetized by inhalation (Isoforine, Cristália^®^, São Paulo, Brazil), and euthanized by exsanguination by cardiac puncture. The tissues were immediately collected, weighted, and frozen in liquid nitrogen, and stored at −80 °C for further analysis. Feces were collected from the cecum, weighted, and stored at −80 °C for future analyses. Colon fragments were collected and fixed in formaldehyde (10% *v*/*v*) for the first 24 h and then stored in ethanol (70% *v*/*v*) and embedded in paraffin for histological analysis.

#### 2.2.4. Histological Analysis

Serial sections of the colon, with a thickness of 5 µm, were collected and subsequently deparaffinized in xylene, rehydrated with different alcohol solutions, and stained with hematoxylin and eosin.

Histological sections were visualized in an Olympus AX70 photomicroscope, and the images were captured in a 20X objective with an AxioCam HRc—Zeiss digital camera. The following features were analyzed: crypt depth, crypt width, and the number of goblet cells. For that, we randomly selected six animals per group and twenty random fields per animal, analyzing one crypt per field. Only crypts with a well-defined and visible structure were used.

The measurements of the crypts were performed using the ImagePro-Plus^®^ version 4.5 software (Media Cybernetics Inc., 1700 Rockville Pike, Suite 240, Rockville, MD 20852, USA) and the goblet cell count was performed using the Image J^®^ 1.48v software (Research Services Branch, National Institute of Mental Health, Bethesda, MD, USA).

#### 2.2.5. Intestinal Permeability

Intestinal permeability analysis was performed at the end of the 10th week of treatment. After 12 h of fasting, the animals received 1 mL of a solution containing 100 mg lactulose and 50 mg mannitol by gavage. Then, they were kept in metabolic cages, fasting for 5 h. The urine was collected for 24 h and stored at −80 °C.

The urine was centrifuged (Hermle centrifuge, model Z326K, Wehingen, Germany), filtered on 0.45 µm membrane filters (Millipore, São Paulo, Brazil), and transferred to vials for high-performance liquid chromatography (HPLC). The mobile phase consisted of water in sulfuric acid (0.005 mM) with an injection volume of 20 µL and a mobile phase flow of 0.6 mL/min [31]. Lactulose^®^ and Mannitol^®^ were used as internal standards (Sigma-Aldrich, São Paulo, Brazil) and the concentrations were transformed to g/L to calculate the percentage of urinary excretion. The lactulose/mannitol ratio was calculated by dividing lactulose concentration by mannitol concentration [32].

#### 2.2.6. Fecal pH and Short-Chain Fatty Acids Analysis

For pH analysis, approximately 1 g of cecum stool was homogenized in 10 mL of distilled water and vortexed with glass beads. Subsequently, the glass electrode of the pH meter was inserted, and the pH was measured in duplicate [33].

The short-chain fatty acids (SCFA) analysis was performed according to Siegfried et al. (1984) with modifications [34]. Briefly, approximately 500 mg of stool samples was homogenized in 1 mL of Milli-Q^®^ water in a vortex and centrifuged at 12,000× *g* for 10 min. The supernatant was removed, and the samples were injected on a high-performance liquid chromatography (HPLC) (injection volume: 20 µL; Dionex Corporation, Sunnyvale, CA, USA). The SCFA were separated on a Phenomenex Rezex ROA ion exclusion column (300 × 7.8 mm) (Phenomenex Inc. Torrance, CA, USA) coupled to a Shodex RI-101 refractive index (IR) maintained at 45 °C. Sulfuric acid 5 mM with a flow of 0.7 mL/min was used as a mobile phase. Stock solutions were prepared using acetic, propionic, and butyric acids as standards with a final concentration of 10 mmol/L (Sigma-Aldrich, Sao Paulo, Brazil). Stock solutions were diluted 2-, 4-, 8-, and 16-fold in 5 mmol/L^−1^ sulfuric acid (0.08–10 mM) to be used as standards in the HPLC analysis.

#### 2.2.7. DNA Extraction and Microbiota Profile

DNA extraction of stool samples collected from the cecum of the animals was performed according to the methodology proposed by Steveron and Weimer (2007) [35]. Briefly, mechanical cellular lysis was performed using glass beads and phenol chloroform to promote the partitioning of lipids and cellular debris into the organic phase. After DNA extraction, a total of 39 samples encompassing all the animals of each experimental group were sequenced at the Argonne National Laboratory, Illinois, USA (AIN-93M n = 10; HFHF: n = 10; GTK: n = 9; and BTK: n = 10). The V4 region of the 16S rRNA genes was amplified by PCR using 515f/806r primers and amplicons sequenced using Illumina MiSeq desktop sequencer producing 150 bp paired-end (PE) reads.

The demultiplexed raw paired-end reads obtained after sequencing were uploaded and processed into QIIME2 (version 2020.2) via the Casava 1.8 paired-end pipeline [36]. DADA2, which allows improved taxonomic resolution based on the exact identification and error correction of sample sequences that differ as little as a single nucleotide, was chosen to assess the quality of the reads in sequential steps such as filtering, trimming, denoising, dereplicating, merging paired reads, as well as chimeric sequences removal [37]. Afterward, amplicon sequence variants (ASV) were forwarded to generate a phylogenetic tree using the align-to-tree-mafft-fasttree pipeline from the q2-phylogeny plugin [38]. When convenient, samples were rarefied to an appropriate sampling depth of 15,349. Taxonomy was assigned to the 16S data using a Naïve Bayes pre-trained Greengenes 13_8 99% OTUs classifier [39].

With regards to the DNA obtained from the kombuchas and their respective SCOBYs, samples used during the experiment were mixed and lyophilized at −62 °C for 24 h under a pressure of 35 uHg (Liotop, model L101, serial no. 01610, Liobras, São Carlos, Brazil). Microbial DNA was extracted from frozen pellets using the Qiagen Powersoil Pro kit with bead beating, according to the manufacturer’s protocol. Then, the samples were forwarded to the company Molecular Research LP (MR DNA, Shallowater, TX, USA) where amplicon preparation and sequencing were performed considering the bacterial V4 region of the 16S rRNA gene (515f/806r primers) and the variable internal transcribed spacer (ITS)-1 of the fungal rRNA region (ITS1F and ITS2 reverse primers). Amplicon libraries were prepared and sequenced with the Illumina MiSeq desktop sequencer producing 250 bp paired-end (PE) reads. For microbiota profiling, sequence data were processed using an MR DNA analysis pipeline (MR DNA, Shallowater, TX, USA). Briefly, sequences were joined, and short reads with ambiguous base calls were removed. Afterward, sequences were quality filtered using a maximum expected error threshold of 1.0, dereplicated, and denoised. Lastly, unique sequences identified with sequencing and/or PCR point errors were removed followed by chimera removal, thereby providing a denoised sequence or zero-radius OTU (zOTU). Final zOTUs were taxonomically classified using BLASTn against a curated database derived from NCBI (http://www.ncbi.nlm.nih.gov; accessed on 10 July 2020). Raw reads were deposited in the Sequence Read Archive (SRA) database (http://www.ncbi.nlm.nih.gov/sra; deposited on 23 November 2022) under the BioProject PRJNA904803.

### 2.3. Statistical Analysis

Statistical analysis was performed using the software GraphPad Prism^®^, version 6.01. The normality of the data was tested by the Shapiro–Wilk test. Groups with parametric distribution were analyzed by one-way ANOVA followed by Tukey post-hoc. Non-parametric data were evaluated by the Kruskal–Wallis test followed by Dunn’s post-hoc. Data were expressed as mean ± standard deviation (SD) and the values were considered significant when *p* < 0.05.

For statistical analysis of the gut microbiota, qiime artifacts were imported into R (R Core Team 3.6.2, 2019) with the qiime2R package v.099.20 (https://github.com/jbisanz/qiime2R; accessed on 10 July 2020). Significant differences in alpha-diversity among the four groups (AIN-93M, GTK, BTK, and HFHF) were determined using the alpha function in microbiome R package v.2.1.24 adopting Kruskal–Wallis as a statistical test followed up by Wilcoxon’s test to calculate pairwise comparisons between groups. For beta-diversity, weighted and unweighted UniFrac distances were subjected to permutational multivariate analysis of variance (PERMANOVA) to assess significant differences (pseudo-F test) in bacterial community composition and structure among the groups with a permutation number of 999. Principal coordinates analysis (PCoA) was chosen to explore and visualize the clustering of groups. All graphs were constructed and visualized with RStudio (v. 1.2.5033) using one or the combination of the following R packages: MicrobiomeR, dplyr, ggplot2, phyloseq [40], tidyr, and vegan.

To determine which bacterial taxa were differentially abundant among groups, taxonomy was firstly collapsed to the genus level and then analyzed via linear discriminant analysis (LDA) effect size (LEFSe) [41] (*p*-value cut-off of 0.05 and log LDA score of 2.0).

## 3. Results

### 3.1. Kombucha Chemical Characterization and Consumption

The main compounds found in kombuchas used in this study are presented in Table 1. As previously demonstrated, both green and black tea kombuchas presented high content of phenolic compounds; however, black tea kombucha presented a higher antioxidant capacity, probably due to its higher phenolic compound concentration [17,25].

Among the 127 phenolic compounds identified in the kombuchas, 14 were more abundant: gallocatechin 3-O-gallate/epigallocatechin 3-O-gallate; gallocatechin isomer 2/epigallocatechin; catechin; quercetin 3-O-rhamnosyl-rhamnosyl-glucoside isomer 2; quercetin 3-O-glucosyl-rhamnosylgalactoside-isomer 2; gallocatechin isomer 1/epigallocatechin; quercetin 3-O-rhamnosyl-rhamnosylglucoside-isomer 1; quercetin 3-O-glucosyl-rhamnosyl-galactoside isomer 1; catechin 3-O-gallate; and catechin 5-O-gallate, which belong to the flavonoids class; and 5-O-galloylquinic acid; 3-[2-(carboxymethyl)-3,4-dihydroxyphenyl] prop-2-enoic acid; 4-coumaroylquinic acid isomer 2; and 1-O-caffeoylquinic acid isomer 2/3-caffeoylquinic acid, which belong to the phenolic acids class [17,25].

**Figure 2:**
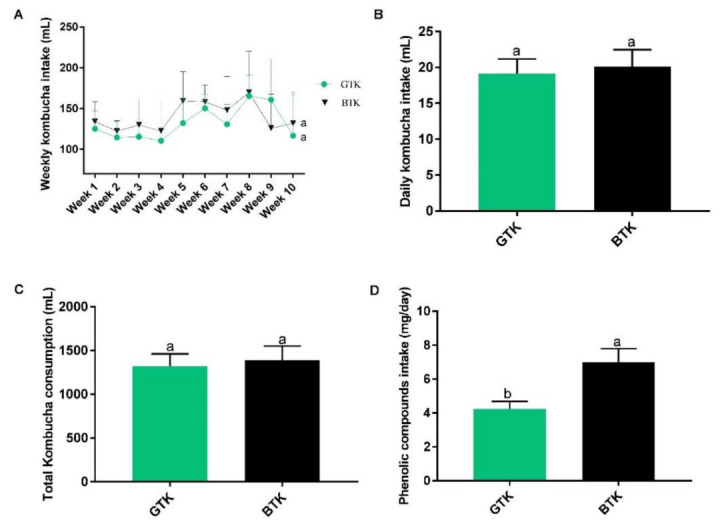
Weekly kombucha intake (**A**), daily kombucha intake (**B**), total kombucha consumption (**C**), and phenolic compounds intake (**D**) by the animals during the treatment. Data were expressed as mean ± SD. Different letters indicate a significant difference (*p* < 0.05) between groups according to t-test. GTK: HFHF diet + green tea kombucha diluted in water (30% *v*/*v*); BTK: HFHF diet + black tea kombucha diluted in water (30% *v*/*v*).

We did not observe a difference between the GTK and BTK groups regarding daily, weekly, and total kombucha consumption. However, the BTK group ingested a higher amount of phenolic compounds, which can be explained by its higher concentration in the black tea kombucha compared to green tea kombucha, as mentioned (Figure 2).

### 3.2. Biometric Parameters

Initial weight, final weight, weight gain, and BMI did not differ among groups, although some metabolic disorders were observed [25]. The group that received a standard diet (AIN-93M) presented a higher cecum weight than the HFHF and GTK groups, but no significant difference was observed compared to the BTK group. Regarding cecum weight:body weight ratio, the AIN93-M group presented a higher value when compared to the GTK group; however, this difference was not significant when compared to the other groups (Table 2).

### 3.3. Bioinformatics Analysis

To better comprehend the effects of the long-term intake of black tea kombucha (BTK) and green tea kombucha (GTK) on the gut microbiota of Wistar rats, we conducted a deep amplicon sequencing of the V4 region of the 16S rRNA genes. After the removal of low-quality and chimeric sequences from 39 datasets (AIN-93M, n = 10; BTK, n = 10; GTK, n = 9; HFHF, n = 10), a total of 1,004,357 high-quality reads, with an average of 25,752 (minimum: 15,349; maximum: 34,422) sequences for each sample, were obtained and assigned to 1218 predicted ASVs (≥99% similarity).

Regarding the sequences obtained from the kombuchas and their respective SCOBYs, 153,322 high-quality reads were obtained based on the amplification of the V4 region of the 16S rRNA genes (mean: 38,330; minimum: 36,957; maximum: 39,631) for bacterial community analysis, whereas 138,569 reads were forwarded for metataxonomic analysis based on the ITS1–2 regions of fungal ribosomal DNA (mean: 34,642; minimum: 34,566; maximum: 34,716).

### 3.4. Microbiota Profiling of GTK and BTK and Their Respective SCOBYs

In both kombuchas and their respective SCOBYs, the fungal community was greatly dominated by the species *Dekkera bruxellensis* (GTK SCOBY: 99.3%; BTK SCOBY: 99.6%; GTK: 99.6%; BTK: 99.9%) and, to a lesser extent, by the species *Saccharomyces bayanus* (GTK SCOBY: 0.7%; BTK SCOBY: 0.4%; GTK: 0.4%; BTK: 0.1%).

**Figure 3:**
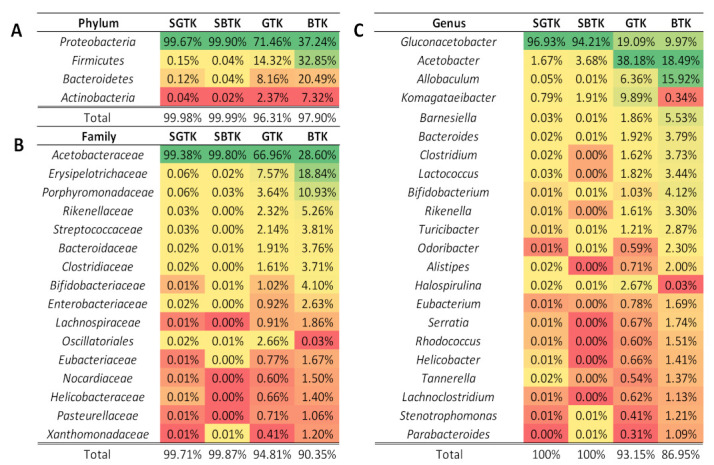
Heat map based on the relative abundance (>1.0% in at least one sample) of the most abundant bacterial taxa of phylum (**A**), family (**B**), and genus (**C**) identified in green tea kombucha (GTK), black tea kombucha (BTK), and their respective SCOBY (SGTK and SBTK). Green to red gradient indicates low to high relative levels of OTUs within the given taxonomic unit.

Regarding the bacterial community, microorganisms belonging to the phylum *Proteobacteria* dominated both tea and SCOBY samples (average of 77.07% considering the four groups); however, in the BTK group, this phylum accounted for only 37.24% of the sequences (Figure 3A). Notably, the phyla *Firmicutes*, *Bacteroidetes*, and *Actinobacteria* also represented an important part of the bacterial community found in kombucha samples, but not in their related SCOBYs. At the family level (Figure 3B), *Acetobacteraceae* stands as the dominant taxon across the groups, corresponding to almost 100% of the samples in the GTK and BTK SCOBYs groups. For kombucha samples, *Acetobacteraceae*, *Erysipelotrichaceae*, *Porphyromonadaceae*, *Rikenellaceae*, and *Streptococcaceae* encompass the top five families. Lastly, at the genus level (Figure 3C), *Gluconacetobacter* appears as the dominant taxon in samples obtained from both SCOBY samples, while *Acetobacter* comprehends the most abundant taxon in kombucha samples. In conjunction with the genus *Acetobacter*, *Allobaculum*, *Komagataeibacter,* and *Barnesiella*, they correspond to the top five genera. Interestingly, kombucha produced from black tea showed a higher number of taxa classified as low-abundant (13.05%), which is quite different from that obtained from the fermentation of green tea (6.85%), which may indicate greater bacterial diversity and richness in the GTK.

### 3.5. Alpha and Beta-Diversity Metrics of Gut Microbiota

**Figure 4:**
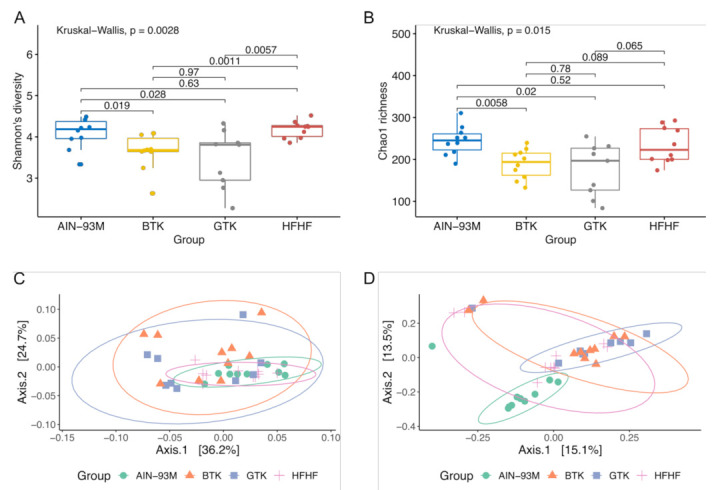
Box and whisker plots comparing species diversity (**A**) and richness (**B**) among the groups AIN-93M, BTK, GTK, and HFHF at the end of the experimental period. Horizontal bold lines show the median values. The bottom and top of the boxes show the 25th and the 75th percentiles, respectively. The whiskers extend up to the most extreme points within 1.5 times the interquartile ranges (IQR). Principal coordinate analysis (PCoA) based on weighted (**C**) and unweighted (**D**) UniFrac distances. PERMANOVA with 999 permutations was used to detect significant differences between microbial communities (dissimilarity) of different experimental groups. Standard error ellipses show 95% confidence areas.

The effects of the daily ingestion of green tea kombucha (GTK) and black tea kombucha (BTK) for 10 weeks on the gut microbiota of Wistar rats were firstly investigated using alpha- and beta-diversity indices and compared against the AIN-93M and HFHF groups. Considering the alpha-diversity analysis, the observed ASVs, Shannon, and Chao1 indices reached a plateau and are indicative that sequencing depth covered most of the microbial diversity and the majority of bacterial phylotypes were sampled (Figure 4).

A significant reduction in bacterial diversity, evaluated through Shannon’s diversity index, was observed in the groups that received green and black tea kombucha when compared to both controls (Figure 4A). However, we did not observe significant differences among the control groups (AIN-93M vs. HFHF), as well as among the treatment groups (GTK vs. BTK) (*p* > 0.05). In terms of bacterial richness, a significantly lower Chao1 index was identified in the groups supplemented with GTK and BTK when compared to the AIN-93M group, but not when compared to the HFHF group (Figure 4B).

Regarding the beta-diversity analysis, gut microbiota clustering on PCoA plots based on weighted and unweighted UniFrac distance metrics showed significant differences among the groups (Figure 4C,D). Pairwise comparisons using Qiime beta-group-significance command revealed that the gut composition of the groups that received the two different types of kombucha significantly differed from those animals receiving a standard (AIN-93M) or a high-fat and high-fructose (HFHF) diet. However, high similarity in terms of bacterial composition and abundance was observed among the GTK and BTK groups. Moreover, according to pairwise PERMANOVA results, the AIN-93M and HFHF groups differed only qualitatively regarding community dissimilarity (unweighted UniFrac: pseudo-F = 4.86, q = 0.0020). Taken together, alpha- and beta-diversity indices evidenced that after receiving an HFHF diet for eight weeks, long-term intake of green and black tea kombuchas was not able to establish a high diversity bacterial community as that observed in both control groups as well as a community as rich as that observed in the AIN-93M group.

### 3.6. Taxonomic Assignment and Gut Bacterial Composition

**Figure 5:**
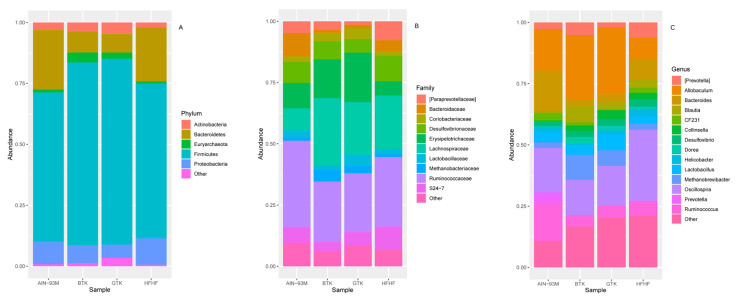
Stacked bar chart based on the relative abundance of major phyla (top-five) (**A**), families (top-ten) (**B**), and genera (top-fifteen) (**C**) across the groups AIN-93M, BTK, GTK, and HFHF.

**Figure 6:**
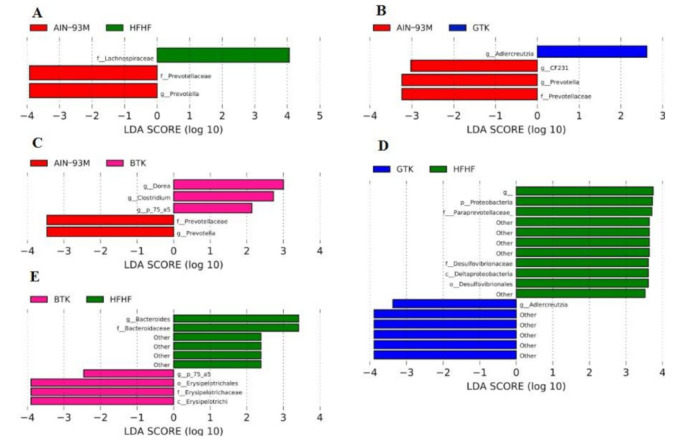
Differential abundance analysis was conducted with Linear discriminant analysis Effect Size (LEfSe) following the experimental period. Comparisons were made between AIN-93M with HFHF (**A**); GTK (**B**); and BTK (**C**) groups and between HFHF with GTK (**D**) and BTK (**E**) groups. Only biomarkers showing linear discriminant analysis (LDA) scores greater than 2.0 with a false discovery rate (FDR) *p* < 0.05 are depicted. Letters: p, phylum; c, class; o, order; f, family; g, genus.

The Linear discriminant analysis Effect Size (LEfSe) was adopted to better characterize the gut bacterial composition of each group, as well as identify the differently abundant taxa. Moreover, inter-group comparisons were conducted based on the relative abundance of interest taxa. At the phylum level, *Firmicutes* (69.09%), *Bacteroidetes* (15.82%), *Proteobacteria* (8.13%), *Actinobacteria* (3.26%), and *Euryarchaeota* (2.15%) were the dominant taxa (Figure 5A). The phylum *Firmicutes* showed higher relative abundance in the groups treated with kombucha prepared from black tea (BTK group, 75.21%) and green tea (GTK, 76.30%) when compared to both control groups (AIN-93M, 61.41%; HFHF, 63.45%), although a significant difference has been observed only between AIN-93M and BTK (*p* = 0.019). The same trend was observed for the phylum *Actinobacteria* (AIN-93M, 2.88%; BTK, 3.56%, GTK, 4.62%; HFHF, 1.97%), although no significant difference among the groups (*p* > 0.05) was observed. On the other hand, *Bacteroidetes* was significantly more abundant in the control groups AIN-93M (24.65%) and HFHF (22.20%) when compared to both kombucha groups (BTK, 8.72%; GTK, 7.69%), but not between AIN-93M and HFHF. Our results also indicate that the administration of both kombuchas increased the *Firmicutes*:*Bacteroidetes* ratio in these groups (BTK: F/B = 8.63; GTK: F/B = 9.93) when compared to the control groups AIN-93M (F/B = 2.49) and HFHF (F/B = 2.86). Regarding *Proteobacteria*, this phylum displayed the lowest proportion in the group GTK (5.38%), whereas its highest relative abundance was observed in the HFHF group (10.86%; Figure 6D, LDA > 3; *p* = 0.035). Interestingly, the phylum *Euryarchaeota* stands out in the BTK group (4.10%); it appears less abundant in the AIN-93M (1.08%) and GTK (2.53%) groups, while a very little abundance was observed in the group HFHF (0.88%) (BTK vs. HFHF, *p* = 0.035).

At the family level, 12 taxa (relative abundance greater than 1%) accounted for approximately 95% of the total sequences in each group. Among them, *Ruminococcaceae* was the most abundant family in the AIN-93M, GTK, and HFHF groups, whereas *Lachnospiraceae* was the most prevalent in the BTK group (Figure 5B). Although *Lachnospiraceae* was the most abundant in the BTK group and differed from the AIN-93M group (*p* = 0.00049), this taxon appears as a biomarker of the HFHF group when compared to AIN93M (Figure 6A, LDA > 3, *p* = 00013). The third most abundant family among the groups, *Erysipelotrichaceae*, appears enriched in the groups that received both kombuchas (AIN-93M, 10.37%; BTK, 15.87%; GTK, 20.21%; HFHF, 5.74%) and was identified as a biomarker of the BTK group when compared to the group HFHF (Figure 6E LDA > 4; *p* = 0.0068). Interestingly, groups treated with green and black kombuchas showed a very low abundance (around 0.1%) of members belonging to the family *Bacteroidaceae*—a biomarker of the group HFHF—when compared to the group BTK (Figure 6E, LDA > 3), and when compared to both control groups (*p* < 0.05), which may justify the higher F/B ratio observed in kombucha treated groups. Positively correlated with diabetes and obesity [42,43], the families *S24–7* and *Desulfovibrionaceae* were significantly less abundant in the BTK and GTK groups, respectively, while in the HFHF group, these families reached their highest values. Indeed, LEfSe analysis revealed that the family *Desulfovibrionaceae*, in conjunction with the family *Paraprevotellaceae*, were detected as biomarkers of the HFHF group when compared to the group that received green tea kombucha (Figure 6D, LDA >3). Lastly, as shown in Figure 6A–C, *Prevotellaceae* was identified as a biomarker of the AIN-93M group (LDA >3) when compared to the other experimental groups enrolled in this study (AIN-93M, 2.42%; BTK, 0.02%, GTK, 0.01%; HFHF, 0.004%).

After defining the most abundant genera, the top-ten taxa were selected and accounted for at least 70% of the total sequences in each group (Figure 5C). *Oscillospira* was the most abundant microorganism in the control group HFHF (*p* < 0.05). In the groups treated with kombuchas, *Allobaculum* was the dominant genus; however, this difference was significant just among the BTK and HFHF groups (*p* = 0.0068). Differential abundance analysis considering taxa at the genus level showed enrichment of *Prevotella* in the AIN-93M group (Figure 6A–C; LDA > 3). Although not considered biomarkers by LefSe analysis, but still included in the top-ten genera, it was possible to identify that *Bacteroides* and *Ruminococcus* are highly abundant in the group AIN-93M when compared to all other groups (*p* < 0.05). Regarding the BTK group, the genera *Dorea*, *Clostridium,* and *p-75-a5*, all of them belonging to the family *Erysipelotrichaceae*, appeared as biomarkers when compared to the AIN-93M group (Figure 6C, LDA > 3). However, when compared to the HFHF group, only the genus *p-75-a5* was identified as a biomarker in the BTK group (Figure 6E, LDA >3). Concerning the GTK, *Adlercreutzia* appeared as a biomarker in this group when compared to both control groups (Figure 6B,D; AIN-93M, LDA >2; HFHF, LDA >3). We did not identify biomarkers between the GTK and BTK groups regardless of the taxonomic level.

**Figure 7:**
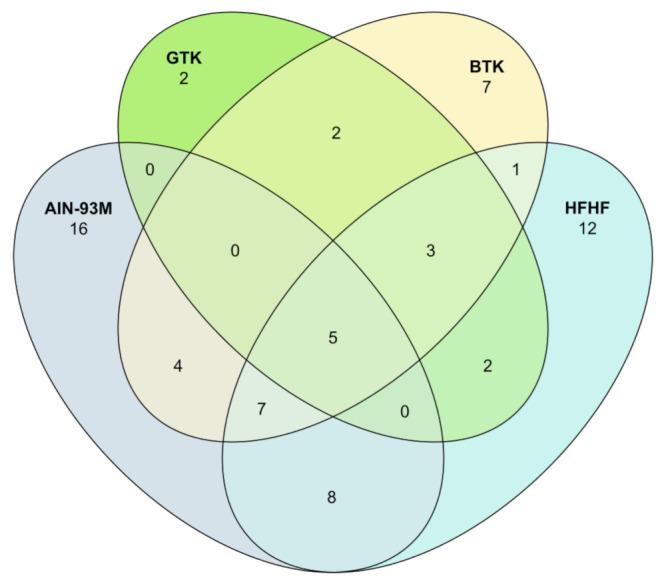
Venn diagram representing shared amplicon sequence variants (ASVs) of the core microbiome identified in the groups AIN-93M, GTK, BTK, and HFHF.

Lastly, we predicted and explored the structural basis (core taxa) of the bacterial communities of the groups enrolled in this study after the experimental period (Figure 7). Considering only ASVs with a prevalence of 75% across samples, the group AIN-93M contained 40 core taxa, while in the groups GTK, BTK, and HFHF, we noticed 14, 29, and 38 taxa, respectively. At a first inspection, five ASVs were identified as common to both groups and assigned to the following taxa: *Clostridiales, Allobaculum,* and *Oscillospira*. Secondly, considering only those ASVs specific to each group, 16 ASVs were identified in samples from the group AIN-93M and assigned to the following taxa: orders *Clostridiales* and *Bacteroidales*, class *Clostridia*, genera *Oscillospira*, *Bacteroides*, and *Helicobacter*, and species *Mucispirillum schaedleri*. Regarding the groups that underwent kombucha ingestion, only two ASVs stood out in the GTK group and were assigned to the genus *Lactobacillus* and the species *Ruminococcus flavefaciens*, whereas seven ASVs were typical for the BTK group and were assigned to the family *Lachnospiraceae*, genera *Dorea*, *Blautia*, *Allobaculum*, and *Mogibacteriaceae*, as well as the species *Collinsella stercoris*. Lastly, 12 ASVs were identified as specifically present in the HFHF group and were taxonomically assigned to the following taxa: families *Desulfovibrionaceae*, *S24-7*, *Ruminococcaceae,* and *Lachnospiraceae*, in addition to the genera *Roseburia* and *Oscillospira*.

### 3.7. Fecal pH and Short-Chain Fatty Acids Content

**Figure 8:**
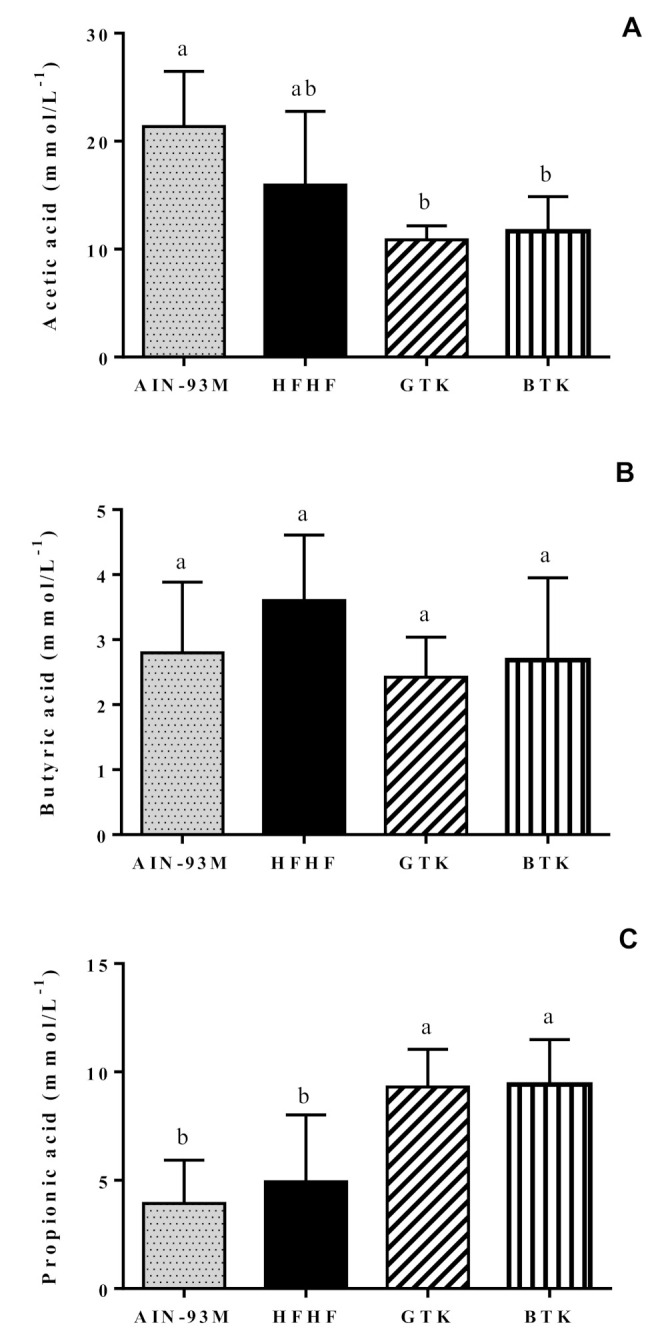
Acetic (**A**), butyric (**B**), and propionic (**C**) acids concentrations identified on stool samples from the animals. AIN-93M: standard diet (negative control group); HFHF: high-fat and high-fructose diet (positive control group); GTK: HFHF diet + green tea kombucha diluted in water (30% *v*/*v*); BTK: HFHF diet + black tea kombucha diluted in water (30% *v*/*v*). Values are expressed as means ± SD. Different letters indicate a significant difference between groups (*p* < 0.05) according to ANOVA one-way followed by Tukey post-hoc (acetic and butyric acids) and Kruskal–Wallis test followed by Dunn’s post-hoc (propionic acid).

We did not find a significant difference in fecal pH among the groups (Table 2). Regarding the SCFA, both treatment groups—GTK and BTK—presented a higher propionic acid concentration when compared to the AIN93-M and HFHF groups. Acetic acid concentration was significantly higher in the AIN93-M group when compared to the GTK and BTK groups, but no significant difference was noted when comparing the HFHF group to the other groups. Butyric acid concentrations did not differ significantly among groups (Figure 8).

We also investigated whether there is an association between the SCFA content and the microorganisms found in the gut microbiota of the animals. For that, MaAsLin 2 [44] was performed to find significant multivariable associations between specific microbial genera, cecal SCFA (acetate, propionate, and butyrate), and phenolic intake. The compound Poisson linear model (CPLM) function was utilized on cumulative sum scaling (CSS) normalized data with minimum prevalence (1%). For analysis of changes across the different interventions, samples obtained from the HFHF group were assigned as references. All *p*-values were false discovery rate-adjusted (Benjamini–Hochberg, q-values), and features with q < 0.25 were considered significant (Table S1).

### 3.8. Intestinal Permeability and Histological Analysis

There was no difference between groups regarding the excretion of lactulose and mannitol in the urine, which was expressed as lactulose/mannitol ratio (Table 2).

**Figure 9:**
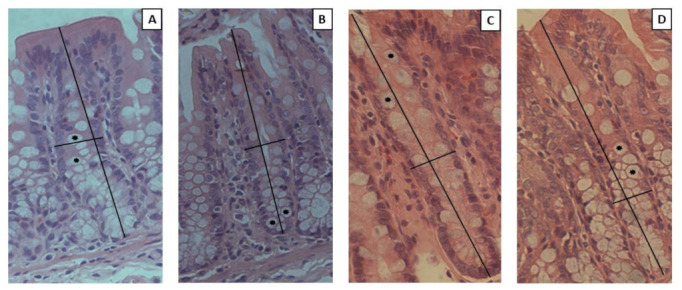
Representative photomicrographs of cecum sections after 10 weeks of treatment. (**A**) AIN-93M; (**B**) HFHF; (**C**) GTK; (**D**) BTK. Vertical lines indicate crypt depth; horizontal lines indicate crypt width; asterisks indicate goblet cells. All images were captured in a 20X objective. AIN-93M: standard diet (negative control group); HFHF: high-fat and high-fructose diet (positive control group); GTK: HFHF diet + green tea kombucha diluted in water (30% *v*/*v*); BTK: HFHF diet + black tea kombucha diluted in water (30% *v*/*v*).

Histological features are demonstrated in Figure 9. We did not observe differences among the groups in terms of crypt depth, crypt width, and the number of goblet cells (Table 2).

## 4. Discussion

In this study, we evaluated the effects of regular kombucha consumption on the gut microbiota and on the intestinal health of Wistar rats fed a high-fat high-fructose diet. Our results show that both green tea (GTK) and black tea (BTK) kombuchas were able to modulate the gut microbiota, which corroborates our hypothesis.

We believe that our results can be attributed, in large part, to the high content and diversity of phenolic compounds present in kombuchas. In general, only a small portion—approximately 5–10%—of the dietary phenolic compound will be absorbed in the small intestine, mainly those with a monomeric or dimeric structure. The more complex ones—oligomeric and polymeric structures—reach the colon practically unchanged where they are metabolized by the gut microbiota, making them more bioactive [45,46,47].

Once biotransformed in less complex compounds such as phenolic acids, the generated metabolites and bioactive molecules will modulate the gut microbiota [45,47,48], exerting an effect similar to prebiotics by favoring the growth of beneficial bacteria and inhibiting the growth of pathogenic ones [47,49]. Studies have shown that the antioxidant and anti-inflammatory activities exerted by the polyphenols act against metabolic disorders such as cancer, obesity, and diabetes via modulation of the gut microbiota [24,48,50,51,52]. There is evidence that metabolic alterations induced by a high-fat diet can also be attenuated by polyphenols intake via activation of PPARα and GLUT4 [53].

Regarding the microbial composition of the kombuchas and their respective SCOBYs, we noticed that the most abundant microorganisms found in the SCOBYs were also found in the beverages, although the diversity was greater in the kombuchas. Interestingly, *Gluconacetobacter* was the predominant genus in both SCOBYs and was much less prevalent in the kombuchas. On the other hand, the genus *Acetobacter* was favored during the fermentation, reaching its maximum abundance in the GTK. Those differences can be explained by the metabolic adaptations of the microorganisms, which are capable of utilizing different substrates depending on the type of tea and consequently will generate different metabolites [17,18,54]. The genus *Acetobacter*, for example, belongs to the group of acetic acid bacteria and has the ability to oxidize ethanol and sugar to acetic acid [55]. Its higher prevalence in the GTK group is probably responsible for the lower pH observed in this beverage.

Our results were partially similar to other studies. Recently, it was evaluated, through metagenomics analysis, the microbial diversity of kombuchas whose fermentation time varied between 3 and 15 days. In all analyzed samples, the bacteria belong to eight phyla, and, likewise to our study, *Proteobacteria* was the predominant one, encompassing more than 99% of the species. Among the yeasts, the genus *Zygosaccharomyces* was predominant (>99%) [20]. In another study, green and black tea kombuchas produced on an industrial scale presented differences in microbiological composition. Lactic acid bacteria, especially *Oenococcus oeni*, was associated with the fermentation of green tea kombucha, while black tea kombucha showed a greater predominance of acetic bacteria. The presence of these bacteria was associated with a higher concentration of lactic acid in green tea kombucha and acetic acid in black tea kombucha. Yeast diversity was not influenced by the type of tea; in both kombuchas, the authors observed a predominance of the species *Dekkera bruxellensis, D. anomala, Hanseniaspora valbyensis,* and *Zygosaccharomyces bailli* [56].

The microbial composition of SCOBYs has also been investigated. In a recent study in which 103 samples obtained from commercial kombucha brewers were analyzed, the authors observed that the microorganisms’ predominance changed according to their position at the SCOBY’s surface. The fungi *Brettanomyces* and the bacteria *Gluconacetobacter*, which have a strong affinity for oxygen, were the main microorganisms found at the upper layer. On the other hand, a greater abundance of *Lactobacillus* was found at the bottom SCOBY side, which corroborates the fact that this genus prefers a low-oxygen environment [21].

Regarding the in vivo results, we should mention that although body composition was not significantly different among the groups, a high-fat and high-fructose diet was able to induce metabolic alterations, as previously reported [15]. As expected, the high-fat content stimulated the growth of gram-negative bacteria such as the phylum *Proteobacteria* and the genus *Bacteroides*, being a biomarker in the HFHF group when compared to the GTK and BTK groups, respectively. Gram-negative bacteria present lipopolysaccharides (LPS) in their cell wall [57], an endotoxin recognized by toll-like receptor 4 (TLR-4) that activates NF-κB (nuclear factor kappa-light-chain-enhancer of activated B cells) and induces the production of pro-inflammatory cytokines such as TNF-α, IL-6, and IL-1β [21]. A higher intestinal permeability allows LPS to migrate into the bloodstream, triggering an inflammatory response in the organism [58]. Our previous work demonstrated that the HFHF diet promoted an increase in the levels of inflammatory markers in the liver (TNF-α) and blood (NLR—neutrophil/lymphocyte ratio) as well as reduced the total antioxidant capacity in plasma and liver and increased oxide nitric concentrations, which were reverted in the groups that consumed both kombuchas [25]. A decrease in the *Proteobacteria* and *Bacteroides* abundance noted in the treatment groups suggest that green and black tea kombuchas may present activity against gram-negative bacteria, which may explain the attenuation of the systemic inflammation and oxidative stress markers observed.

The HFHF diet also favored the *Lachnospiraceae* family, which was a biomarker in this group when compared to AIN93-M, but not when compared to both treatments. Although bacteria that belong to the *Lachnospiraceae* family usually promote a good impact on the gut microbiota by being involved in SCFA production [59,60], it can also be a result of the bile acids’ metabolism. The liver is the main organ responsible for lipid metabolism, where the primary bile acids are synthesized from cholesterol [61,62]. The liver–gut axis is not completely understood yet, but it is known that these bile acids can be used as substrates by the microorganisms in the colon and are converted into secondary bile acids, which are pro-inflammatory metabolites involved in steatosis and NAFLD (non-alcoholic fatty liver disease) [62,63]. Studies conducted with humans [64] and mice [65] have pointed out that the main microorganisms involved in this mechanism belong to the *Lachnospiraceae* family, particularly *Blautia* and *L. incertae sedis* [60,66], and elevated taxa of these bacteria in the gut microbiota have been related to liver diseases. Indeed, as reported in our previous work [25], the HFHF diet has induced liver steatosis in the animals, which was reverted from degree 2 to 1 in those treated with both kombuchas.

The genus *Prevotella* and its family *Prevotellaceae* were identified as biomarkers in the AIN-93M group when compared to the other groups, especially HFHF. They belong to the *Bacteroidetes* phylum and are involved in the metabolism of complex carbohydrates and cellulose. De Filippo et al. (2010) compared the gut microbiota of children living in a rural area in Africa who followed a low-fat and high-fiber diet versus children living in an industrialized city in Europe, whose diet has a high content of fat and protein. The African children presented a higher abundance of the genus *Prevotella* and a higher content of SCFA, which were attributed to a healthier diet [67]. *Erysipelotrichaceae* species are also related to diets rich in carbohydrates and negatively correlated with *Prevotella*. The genera *Dorea*, *Clostridium*, and *p-75-a5*, all belonging to the family *Erysipelotrichaceae*, were reported as biomarkers in the BTK group when compared to the AIN-93M group. The genus *Dorea* has been positively correlated to a Western diet [68] and *p-75-a5* is involved in protein and lipid digestion [69]. Interestingly, when comparing the BTK and HFHF groups, the genus *p-75-a5* and its family *Erysipelotrichaceae* were negatively associated with the BTK group, suggesting that BTK consumption was able to attenuate the negative impact of a HFHF diet. The same biomarkers were not observed in the GTK group, although it also presented a high abundance of the *Erysipelotrichaceae* family.

Considering the treatment groups, we observed that *Actinobacteria* was present in a higher amount in both of them. This phylum is associated with short-chain fatty acids (SCFA) production [70], which are by-products derived from microbial fermentation that are used as an energy source by the enterocytes, favoring intestinal homeostasis and metabolism [70,71]. SCFA also act in the activation of the hormones GLP-1 (glucagon-like peptide 1) and PYY (peptide YY). GLP-1 regulates appetite by inhibiting gastric emptying and stimulating insulin secretion. PYY, in turn, is involved in appetite reduction and gastric motility inhibition. Thus, SCFA act by regulating food consumption and satiety [72], acting on obesity control. Recent studies have suggested that SCFA can modify the epigenome, acting on tissues and organs besides the intestine [71]. Beyond *Actinobacteria*, other microorganisms are pointed out as SCFA producers as those belonging to the *Bifidobacterium* and *Lactobacillus* genera. Both have shown an increased abundance in treatment groups, while *Lactobacillus* was especially higher in the GTK group.

Among SCFA, butyrate is considered the main energy source of colonocytes and enterocytes, thus favoring their growth [73,74]. In our study, butyrate concentrations did not differ significantly among the groups. On the other hand, we observed a higher acetate production in the AIN-93M and HFHF groups, although this last one was not significantly different from the treatments. Since acetate is a metabolite produced especially by bacteria from the *Bacteroidetes* phylum [71], it may explain its elevated abundance in those groups. Finally, we observed a higher concentration of propionate in both treatment groups. This SCFA is produced by a few bacteria, especially those from the genus *Akkermansia*. Phenolic compounds can induce changes in microbial composition, favoring the growth of *Akkermansia muciniphila* [75]. Since both kombuchas present high amounts of phenolic compounds [17,76], it may explain the increase in the *Akkermansia* abundance observed in the treatment groups. The presence of *Akkermansia muciniphila* is associated with a decrease in intestinal permeability and beneficial effects on diabetes mellitus and obesity [77,78].

In both treatment groups, we also observed the presence of *Ocillospira*, a genus involved in glucuronic acid degradation that is positively associated with leanness and health [79]. However, its presence was predominant in the HFHF group, probably because *Ocillospira* can use metabolic products secreted by other microorganisms, including *Bacteroides*. Since a high-fat diet stimulates the growth of *Bacteroides*, it can indirectly favor *Ocillospira* [79].

Finally, when considering the LEfSe analysis, the genus *Adlercreutzia* was observed as a biomarker in the GTK group when compared to both controls. This genus, and more particularly, *Adlercreutzia equolifaciens*, exerts a fundamental role in the metabolism of polyphenols in conjunction with other bacteria such as *Flavonifractor plautii, Slackia equolifaciens, Slackia isoflavoniconvertens, Eubacterium ramulus, Eggerthella lenta,* and *Bifidobacterium* spp. [45].

Our study has several strengths. To our knowledge, this is the first that investigated the effects of regular kombucha consumption on the intestinal health of rats fed a high-fat high-fructose diet. Our methodology allowed us to compare both kombuchas and sequencing the beverages and their respective SCOBYs was crucial to analyze if those results reflect the ones found in vivo. The results will help on the understanding of the mechanisms involved in kombucha consumption, and certainly will contribute to filling out the lack of evidence about its impact on intestinal health. As the main limitation, we should mention that the gut microbiota analyses were performed using stool samples from the cecum, which probably has not allowed us to fully explore the results in the same way as if they were collected after undergoing the whole large intestine. The literature is still limited, and the results are controversial, so more studies are necessary to confirm those hypotheses.

## 5. Conclusions

Our results demonstrated that diets were able to modulate the gut microbiota in different ways. A high-fat high-fructose diet, as expected, was associated with the prevalence of pathobionts, such as *Proteobacteria* and *Bacteroides*. Even though a healthier diet will be always encouraged to prevent and attenuate metabolic disorders, we have noticed that kombucha intake could somewhat modulate the gut microbiota, mitigating the impairments provoked by a Western diet by increasing propionate production and favoring the growth of beneficial bacteria, such as *Adlercreutzia* in the GTK group. Thus, we conclude that regular kombucha intake may be beneficial to intestinal health, although more studies, especially clinical trials, are necessary.

## Figures and Tables

**Table 1:** Green and black tea kombuchas chemical characterization.

	Green Tea Kombucha	Black Tea Kombucha	*p*-Value
**Chemical composition**			
Sucrose (g/L)	19.30 ± 2.73 ^b^	34.98 ± 1.42 ^a^	0.0382
Glucose (g/L)	3.19 ± 0.15 ^a^	2.45 ± 0.96 ^a^	0.4690
Fructose (g/L)	0.15 ± 0.01 ^a^	0.05 ± 0.02 ^a^	0.0583
Ethanol (g/L)	7.23 ± 0.03 ^a^	4.91 ± 0.35 ^a^	0.0653
Theaflavin (g/L)	0.28 ± 0.03 ^b^	1.51 ± 0.06 ^a^	0.0066
Thearubigin (g/L)	13.30 ± 0.67 ^b^	19.99 ± 0.10 ^a^	0.0416
pH	3.2 ± 0.1 ^b^	3.5 ± 0.1 ^a^	0.0078
Total acidity (% *w*/*v*)	0.36 ± 0.01 ^a^	0.32 ± 0.01 ^b^	0.0100
**Organic acids**			
Acetic acid (g/L)	3.22 ± 0.39 ^a^	2.78 ± 0.16 ^a^	0.3336
Glucuronic acid (g/L)	1.17 ± 0.06 ^a^	0.47 ± 0.02 ^b^	0.0323
Lactic acid (g/L)	0.01 ± 0.00 ^a^	0.02 ± 0.00 ^a^	0.2604
**Microbiological characterization**			
Acetic bacteria (log CFU/mL)	6.0 ± 0.30 ^a^	5.30 ± 0.10 ^a^	0.1071
Lactic bacteria (log CFU/mL)	6.50 ± 0.20 ^a^	5.90 ± 0.60 ^a^	0.3959
Yeast (log CFU/mL)	6.30 ± 0.40 ^a^	5.50 ± 0.10 ^a^	0.1690

Values are expressed as mean ± SD. Different letters in the same row indicate a significant difference (*p* < 0.05) according to unpaired *t* test followed by Welch’s correction. Details about the methodology used for the analyses are described in Cardoso et al. (2020).

**Table 2:** Body composition and intestinal parameters of the animals after 10 weeks of treatment.

Features	AIN93-M (n = 10)	HFHF (n = 10)	GTK (n = 9)	BTK (n = 10)
**Body composition**				
Initial weight (g)	349.90 ± 30.71 ^a^	366.90 ± 36.90 ^a^	370.40 ± 36.20 ^a^	364.60 ± 36.05 ^a^
Final weight (g)	415.00 ± 34.50 ^a^	438.10 ± 66.65 ^a^	415.30 ± 37.07 ^a^	409.60 ± 50.08 ^a^
Weight gain (g)	65.00 ± 22.70 ^a^	71.25 ± 38.10 ^a^	44.90 ± 30.69 ^a^	44.90 ± 23.14 ^a^
BMI (g/cm^2^)	0.68 ± 0.08 ^a^	0.61 ± 0.04 ^a^	0.61 ± 0.08 ^a^	0.61 ±0.08 ^a^
Cecum weight (empty) (g)	1.01 ± 0.23 ^a^	0.97 ± 0.13 ^a^	0.96 ± 0.18 ^a^	0.96 ± 0.04 ^a^
Cecum weight (full) (g)	5.09 ± 1.15 ^a^	3.92 ± 0.89 ^b^	3.62 ± 0.75 ^b^	4.03 ± 0.80 ^ab^
Cecum weight:body weight ratio	1.23 ± 0.28 ^a^	0.91 ± 0.24 ^ab^	0.87 ± 0.17 ^b^	0.98 ± 0.21 ^ab^
**Intestinal Permeability**				
Lactulose:mannitol ratio	1.51 ± 0.57 ^a^	1.56 ± 0.78 ^a^	1.62 ± 0.94 ^a^	2.17 ± 1.08 ^a^
**Histological Features**				
Crypt depth (µM)	179.10 ± 43.20 ^a^	223.10 ± 40.69 ^a^	221.20 ± 24.92 ^a^	209.30 ± 40.83 ^a^
Crypt width (µM)	19.51 ± 2.66 ^a^	18.85 ± 4.16 ^a^	21.03 ± 1.46 ^a^	19.46 ± 2.68 ^a^
Number of goblet cells (units)	18.60 ± 3.27 ^a^	16.59 ± 4.71 ^a^	17.94 ± 2.93 ^a^	22.38 ± 5.51 ^a^
**Fecal pH**	9.01 ± 0.40 ^a^	9.17 ± 0.25 ^a^	9.27 ± 0.07 ^a^	9.13 ± 0.13 ^a^

Data are expressed as mean ± SD. Different letters in the same row indicate a significant difference (*p* < 0.05) according to one-way ANOVA followed by Tukey post-hoc (parametric data) or Kruskal–Wallis test followed by Dunn’s post-hoc (non-parametric data). AIN-93M: standard diet (negative control group); HFHF: high-fat and high-fructose diet (positive control group); GTK: HFHF diet + green tea kombucha diluted in water (30% *v*/*v*); BTK: HFHF diet + black tea kombucha diluted in water (30% *v*/*v*).

## Data Availability

Not applicable.

## Supplementary Materials

The following supporting information can be downloaded at: https://www.mdpi.com/article/10.3390/nu14245234/s1. Table S1. Multivariable associations between specific microbial genera, fecal short-chain fatty acids, and phenolic intake.
